# Mica Nanoparticle, STB-HO Eliminates the Human Breast Carcinoma Cells by Regulating the Interaction of Tumor with its Immune Microenvironment

**DOI:** 10.1038/srep17515

**Published:** 2015-12-03

**Authors:** Tae-Wook Kang, Hyung-Sik Kim, Byung-Chul Lee, Tae-Hoon Shin, Soon Won Choi, Yoon-Jin Kim, Hwa-Yong Lee, Yeon-Kwon Jung, Kwang-Won Seo, Kyung-Sun Kang

**Affiliations:** 1 Adult Stem Cell Research Center, College of Veterinary Medicine, Seoul National University, Seoul 151-742, South Korea; 2 Research Institute for Veterinary Medicine, College of Veterinary Medicine, Seoul National University, Seoul 151-742, South Korea; 3 Institute for Stem Cell and Regenerative Medicine in Kangstem Biotech, Biomedical Science Building, College of Veterinary Medicine, Seoul National University, Seoul 151-742, South Korea; 4 The Faculty of Liberal Arts, Jungwon University, Chungbuk, 367-805, South Korea; 5 Seobong BioBestech Co., Ltd., Yeoksam-dong, Kangnam-gu, Seoul, South Korea

## Abstract

Mica, an aluminosilicate mineral, has been proven to possess anti-tumor and immunostimulatory effects. However, its efficacy and mechanisms in treating various types of tumor are less verified and the mechanistic link between anti-tumor and immunostimulatory effects has not been elucidated. We sought to investigate the therapeutic effect of STB-HO (mica nanoparticles) against one of the most prevalent cancers, the breast cancer. STB-HO was orally administered into MCF-7 xenograft model or directly added to culture media and tumor growth was monitored. STB-HO administration exhibited significant suppressive effects on the growth of MCF-7 cells *in vivo*, whereas STB-HO did not affect the proliferation and apoptosis of MCF-7 cells *in vitro*. To address this discrepancy between *in vivo* and *in vitro* results, we investigated the effects of STB-HO treatment on the interaction of MCF-7 cells with macrophages, dendritic cells (DCs) and natural killer (NK) cells, which constitute the cellular composition of tumor microenvironment. Importantly, STB-HO not only increased the susceptibility of MCF-7 cells to immune cells, but also stimulated the immunocytes to eliminate cancer cells. In conclusion, our study highlights the possible role of STB-HO in the suppression of MCF-7 cell growth via the regulation of interactions between tumor cells and anti-tumor immune cells.

Breast cancer is one of the most prevalent cancers observed in women, with high incidence and mortality rates. Annually, approximately 1.38 million women worldwide are diagnosed with this disease, which is the second leading cause of cancer-related deaths^1^. The most common types of cancer treatment include surgery, chemotherapy, radiation therapy and immunotherapy^2^,^3^,^4^. However, the primary treatment is based on chemotherapy, which still has the issues of systemic toxicity and drug resistance. These therapeutic limitations led researchers to develop targeted cancer therapies. Drugs or other natural compounds have been developed as targeted therapy for cancer to suppress the proliferation and metastasis of cancer cells by specifically blocking crucial molecules or pathways with little damage to normal cells^3^. Therefore, several natural compounds such as plant extracts, minerals, vitamins or the combination of these compounds, have been suggested as alternative anti-tumor medicines^5^,^6^,^7^.

Mica has been reported to have the anti-tumor and the immunostimulatory effects. A recent study has demonstrated that mica exhibits the chemopreventive potential against colorectal cancers^8^. Moreover, mica group has been used as feed supplements to enhance immune activity due to its ability to stimulate immune responses against virus infection^9^,^10^,^11^. Recently, Jung *et al*. showed that mica nanoparticles activate macrophages via the up-regulation of lysosome and phagosome pathway^12^. However, although the immunostimulatory ability can be efficiently used to suppress tumor, most studies using mica have investigated the anti-tumor and the immunostimulatory effects separately, but none have tried to integrate these effects by elucidating mechanistic action of mica on immune cells constituting tumor microenvironment. In addition, till now, a study regarding the anti-tumor effect of mica has been performed against only one type of cancer, even though more than 100 types of cancers have been isolated and characterized including breast cancer cells^13^.

Natural Killer (NK) cells are innate immune cells that play a crucial role in antitumor response by exerting strong cytotoxic activity against cancer cells^14^. Upon activation, NK cells eradicate target cells through the secretion of cytolytic enzymes or cytokines including interferon (IFN)-γ^15^,^16^. Moreover, the crosstalk of NK cells with other immune cells is important for anti-tumor responses. For example, DCs can activate resting NK cells, which in turn, induce DC maturation^17^,^18^,^19^. Recently, it has been demonstrated that macrophages also regulate the anti-tumor responses of NK cells via bidirectional interactions^20^,^21^,^22^. Bellora *et al*. reported that immunostimulatory type of macrophages (M1) induces strong activation of resting NK cells and the polarization of macrophages toward M1 phenotype can restore immunomodulatory type of macrophages (M2), the general phenotype in cancer tissues^23^. These findings indicate that the crosstalk between NK cells and other immune cells might be an important target of mica to promote anti-tumor immunity. Therefore, the present study was designed to investigate the anti-tumor effect of STB-HO (mica nanoparticles) against MCF-7, the human breast carcinoma cell line. Furthermore, in an attempt to verify the underlying mechanisms, we explored the direct effects of STB-HO on MCF-7 as well as the indirect, immunity-mediated effects.

## Results

### STB-HO reduces tumor growth in MCF-7 xenograft model

**Figure 1:**
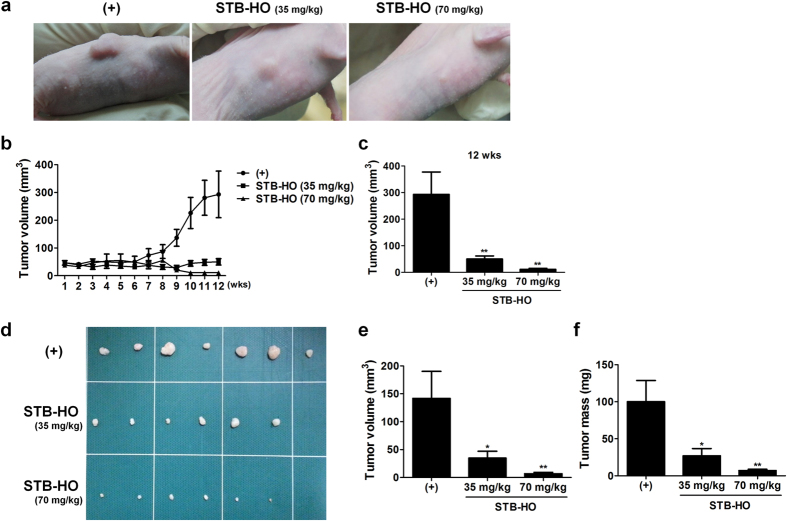
Suppressive effect of STB-HO on MCF-7 growth in athymic nude mice. (**a–c**) From 1 week of stabilization period after MCF-7 xenotransplantation, STB-HO was orally administrated daily in two different dose (35 mg/kg or 70 mg/kg) for 12 weeks and tumor size was measured twice a week. (**a**) Photographs of tumors in mice. (**b**) Tumor volume measurement in a time course. (**c**) Tumor volume at 12 weeks. (**d–f**) Tumors from xenograft mouse model were isolated and measured. (**d**) Photographs of isolated tumors. (**e**) Volume and (**f**) Mass of dissected tumors. Seven to eight mice per group were used. *P < .05, **P < .01. Results are shown as mean ± SD.

To validate anti-cancer effects of Mica, we investigated the impact of STB-HO on breast cancer cell growth in mouse xenograft model. MCF-7 cells were subcutaneously transplanted into athymic nude mice. After 1 week, two different concentrations of STB-HO were orally administered into mice for 12 weeks and tumor volume was measured twice a week. Daily administration of STB-HO did not exert any adverse effects such as body weight loss, abnormal behavior and sudden death, indicating that the compound does not have toxicity *in vivo*. We observed that the tumor size did not as much grow in both low- and high-concentration of STB-HO-treated mice as normal saline-treated mice (Fig. 1a,b). After 12 weeks of treatment, tumor volume in STB-HO-administered group was significantly lower compared to normal saline-treated group (Fig. 1c). Consistently, the volume and the mass of isolated tumors from sacrificed mice were significantly suppressed by STB-HO treatment in a dose dependent manner (Fig. 1d–f).

**Figure 2:**
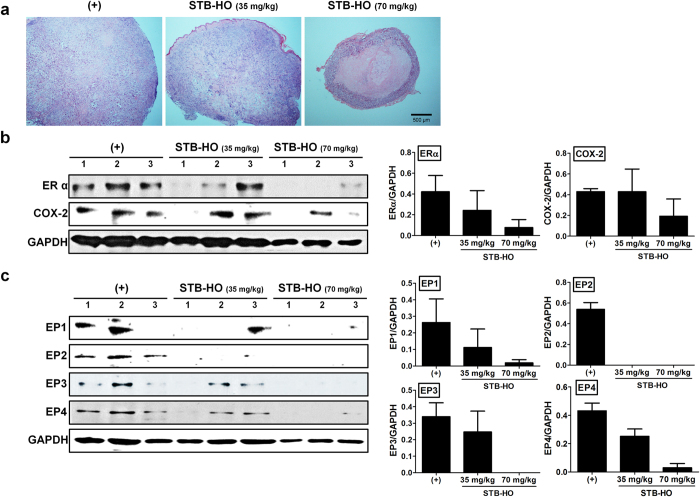
Regulation of crucial signaling in MCF-7 tumor formation by STB-HO. Dissected tumors were processed for further histological analysis and specific protein detection. (**a**) Isolated tumors were stained with hematoxylin and eosin. (**b,c**) Tumors were lysed and determined for the expression of crucial proteins in tumor growth. (**b**) ERα and COX-2 expression in tumor samples were detected by western blotting and quantified. (**c**) Expressions of four EP receptors were determined and quantified. Results are shown as mean ± SD.

Upon histological examination of isolated tumor, massive infiltration of lymphocytes was observed at marginal location of tumor tissue from high concentration STB-HO-treated group, implying the potential of immunostimulatory effects by STB-HO (Fig. 2a). As estrogen receptor α (ERα), prostaglandin E_2_ (PGE_2_) produced by cyclooxygenase-2 (COX-2) and individual E-series of prostaglandin (EP) receptors are known to play critical roles in breast cancer cell pathogenesis, progression, malignancy and metastasis^24^,^25^, we assessed the expression of each protein in tumor tissue. We observed the dose-dependent reduction in the expression of ERα in tumor from STB-HO-treated group. Furthermore, treatment of high dose STB-HO led to the decrease in COX-2 expression (Fig. 2b). STB-HO treatment also down-regulated the expression of four types of receptor for PGE_2_, designated as EP 1–4 (Fig. 2c). We next explored the distribution of STB-HO in tumor tissue using the specific staining for aluminum, one of the STB-HO constituents. However, we couldn’t detect any significant difference in the staining of tumor sections from STB-HO-administered group compared to normal saline-treated group (Suppl. Fig. S1). Taken together, these results indicate that STB-HO administration can suppress the tumor growth and the expression of crucial factors for tumor progression and metastasis.

### STB-HO does not directly regulate the proliferation and viability of MCF-7 cells

**Figure 3:**
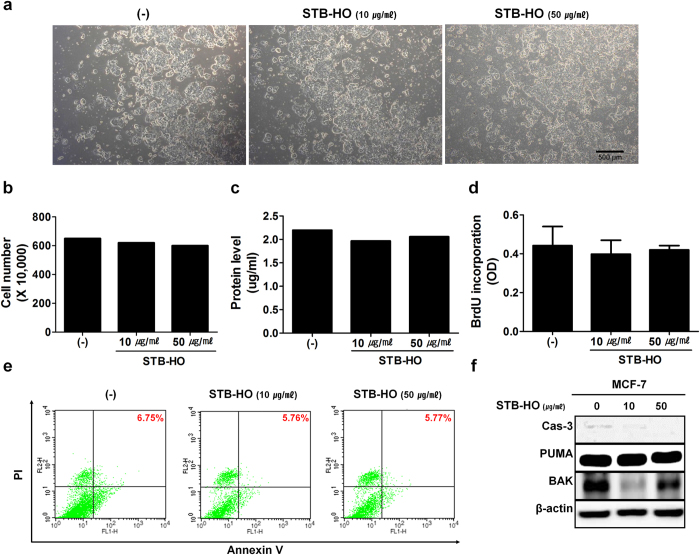
Direct effects of STB-HO treatment on MCF-7 cell proliferation and apoptosis. (**a**) MCF-7 cells were treated with two different concentrations of STB-HO for 3 days and photographs were taken. (**b–d**) MCF-7 proliferation in response to STH-HO was determined with various methods (**b**) A trypan blue exclusion test was performed. (**c**) Concentration of total protein from lysed cell was measured. (**d**) BrdU incorporation assay was performed. (**e**) Apoptotic rate in MCF-7 cells was measured by staining Annexin V after STB-HO treatment. (**f**) Apoptosis-related proteins were detected by western blot analysis. Results are one representative experiment of three independent experiments. Results are shown as mean ± SD. Cas-3; caspase-3, PUMA; p53 upregulated modulator of apoptosis, BAK; Bcl-2 homologous antagonist/killer.

To verify the mechanism of STB-HO action on breast cancer cells, we investigated whether treatment of STB-HO can affect the proliferation or apoptosis of various cancer cells including MCF-7 cells. Phagocytosis of STB-HO particles by cancer cells was detected by time-lapse microvideography (Suppl. Video S1). After 72 hours of treatment, STB-HO inhibited the proliferation of cancer cells including prostate cancer (DU145 and PC3), glioblastoma (U87) and breast cancer (MDA-MB-231), whereas unexpected slight inhibition was observed in the proliferation of MCF-7 cells when same concentration of STB-HO was treated (Figure 3a–d and Suppl. Fig. S2). Similarly, STB-HO treatment didn’t induce the apoptosis of MCF-7 cells nor increase the expression of apoptosis-related signals while those signals were activated in prostate cancers and glioblastoma in response to STB-HO (Fig. 3e,f and Suppl. Fig. S2). On the other hand, to evaluate the *in vitro* toxicity of STB-HO, we treated STB-HO on human dermal fibroblasts and observed that the proliferation or the apoptosis of fibroblasts were not affected by the treatment (Suppl. Fig. S2). These findings suggest that STB-HO does not have direct effect on the growth of MCF-7 cells, and that indirect mechanisms might be involved in the anti-tumor effect of STB-HO in xenograft model.

### STB-HO increases the susceptibility of MCF-7 cells to their microenvironment

**Figure 4:**
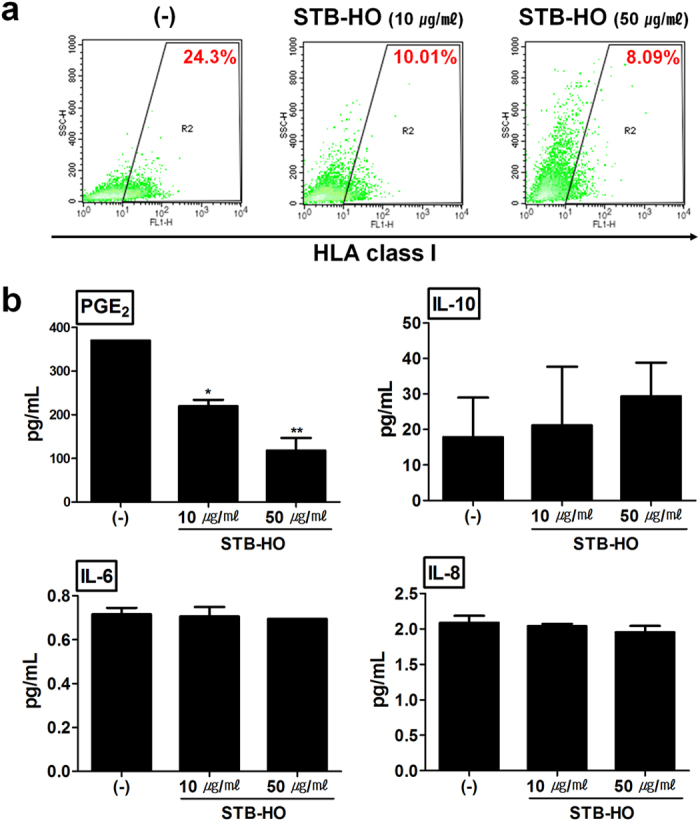
Regulation of immune evasive components in MCF-7 cells by STB-HO treatment. (**a**) The expression of HLA class I on the surface of MCF-7 cells was detected by flow cytometry after STB-HO treatment. (**b**) The secretion of soluble factors related with immune evasion was measured using commercial kits. Production of PGE_2_, IL-10, IL-6 and IL-8 from MCF-7 cells was detected from culture supernatant. *P < .05, **P < .01. Results are one representative experiment of three independent experiments. Results are shown as mean ± SD.

Given that the anti-tumor effect of STB-HO might involve indirect mechanisms rather than direct inhibitory effect on cancer cell growth, we next examined whether STB-HO can regulate the evasive behavior of MCF-7 to avoid the attack by immune system. HLA class I molecule is a well-known inhibitory factor for NK cell-mediated anti-tumor effect. Accordingly, it has been reported that NK cells can kill target cells expressing low levels of HLA class I molecule^26^. Therefore, we first examined the alteration in the expression of HLA class I molecule by STB-HO treatment. Interestingly, while 24% of MCF-7 cells expressed MHC-expressed class I antigens, HLA-ABC, on cell surface, STB-HO treatment down-regulated the expression of these antigens to approximately 10% (Fig. 4a). This inhibitory effect of STB-HO on the expression of MHC class I was consistently observed in other types of cancer cells (Suppl. Fig. S3). In addition, because tumor cells are reported to use immunomodulatory soluble factors such as IL-6, IL-8, IL-10 and PGE_2_ for immune evasion^27^,^28^,^29^,^30^,^31^, we next detected the concentration of these soluble factors secreted by MCF-7 cells after STB-HO treatment. The concentration of PGE_2_ in MCF-7 culture media was significantly reduced by STB-HO treatment in a dose-dependent manner, whereas other cytokines were hardly detectable (Fig. 4b). Taken together, these results indicate that STB-HO treatment attenuates the immune evasive ability of MCF-7 cells by impairing their production of inhibitory factors.

### STB-HO skews macrophages and dendritic cells toward anti-tumor type

**Figure 5:**
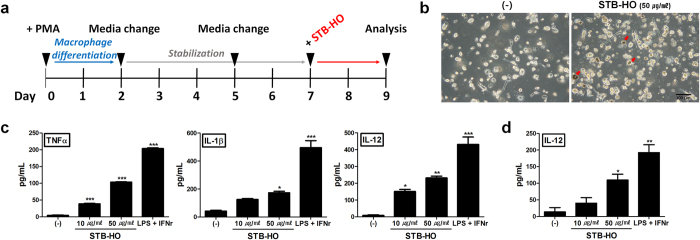
Polarization of macrophages by STB-HO treatment. (**a**) A time line for the differentiation of THP-1 into macrophage-like cells. (**b**) Photographs of THP-1-derived macrophages after STB-HO treatment. STB-HO engulfed by macrophages is indicated by red arrow. (**c**) TNF-α, IL-1β and IL-12 secretion was measured in culture media of THP-1-derived macrophages. (**d**) Production of IL-12 from MNC-derived macrophages was detected. Treatment of LPS with IFN-γ was used to induce the polarization of macrophages. *P < .05, **P < .01, ***P < .001. Results are one representative experiment of three independent experiments. Results are shown as mean ± SD.

**Figure 6:**
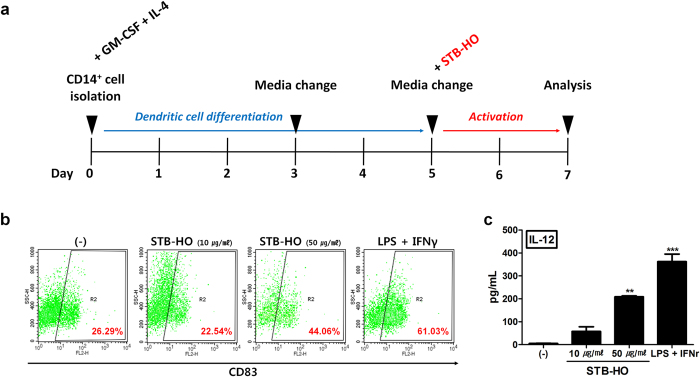
Activation of DCs by STB-HO treatment. (**a**) Outline for DC induction from human MNCs. (**b**) The expression of CD83, DC activation marker, was detected by flow cytometry after STB-HO treatment. (**c**) IL-12 concentration in culture media of DCs was measured. Treatment of LPS with IFN-γ was used to induce the maturation of DCs. **P < .01, ***P < .001. Results are one representative experiment of three independent experiments. Results are shown as mean ± SD.

Since we found that STB-HO increased the susceptibility of MCF-7 cells to immune cells, we then investigated the effects of STB-HO on major cells of anti-tumor immunity. We first analyzed the functional alteration in macrophages and dendritic cells in response to STB-HO. Macrophage-like cells were induced from THP-1, followed by STB-HO treatment (Fig. 5a). We observed that macrophages engulfed STB-HO (Fig. 5b) and their secretion of TNF-α, IL-1β and IL-12, prominent cytokines of M1 type macrophage, were augmented by STB-HO treatment (Fig. 5c). Among these cytokines, IL-12 is known to critically contribute to the NK cell cytotoxicity against tumor cells. Therefore, we confirmed the consistent increase in IL-12 production by STB-HO treatment using primary cultured macrophages (Fig. 5d). DCs were prepared from CD14^+^ monocytes and determined for STB-HO-induced activation (Fig. 6a). To analyze the activation of DCs, we stained cells with CD83, one of the surface markers indicating DC activation. We observed that high concentration of STB-HO increased CD83 expression up to 44% compare to 26% in naïve DCs (Fig. 6b). Consistently, IL-12 secretion of DCs in response to STB-HO was enhanced (Fig. 6c). However, low concentration of STB-HO did not alter the expression of DC activation marker, implying that appropriate dose of STB-HO for the activation of macrophages or DCs might be distinct.

### STB-HO up-regulates the NK cell-mediated killing of MCF-7 cells *in vitro* and increases the number of NK cells *in vivo*

**Figure 7:**
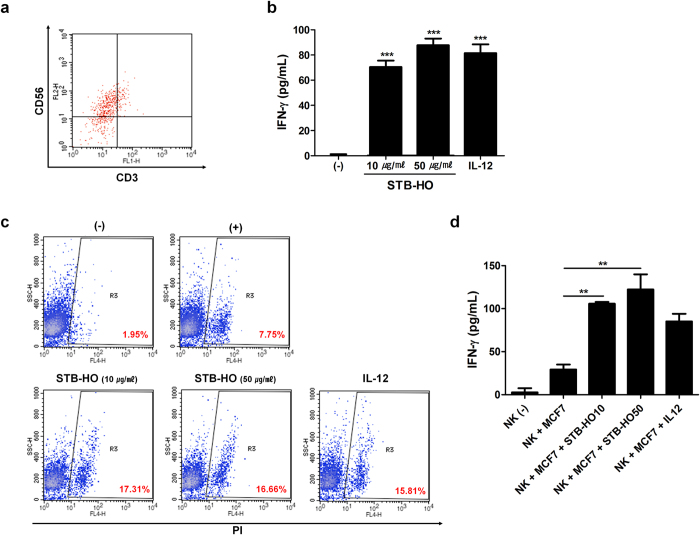
Enhancement of NK cell cytotoxicity upon STB-HO treatment. (**a**) Isolated NK cells were determined for the purity through the detection of CD56 and CD3 on cell surface using flow cytometry. CD3^-^ CD56^+^ cells represent NK cells. (**b**) IFN-γ  secretion from isolated NK cells was measured. (**c,d**) CFDA-labeled MCF-7 cells were co-cultured with NK cells in the presence of STB-HO for 4 hours. (**c**) Co-cultured cells were stained with PI and PI-expressing dead cells were detected by flow cytometry. (**d**) Culture media used in co-culture was determined for the production of IFN-γ. IL-12 treatment was used to induce the activation of NK cells. **P < .01, ***P < .001. Results are one representative experiment of three independent experiments. Results are shown as mean ± SD.

To explore the effect of STB-HO on NK cells, a major component of the anti-tumor immunity, we isolated NK cells from human blood and evaluated the purity of isolated cells by targeting CD3^-^ CD56^+^ cell population (Fig. 7a). After STB-HO treatment, the secretion level of IFN-γ from isolated NK cells was significantly elevated to the extent that was observed in cells activated by IL-12 as a positive control group (Fig. 7b). To better demonstrate the actual cytotoxicity of activated NK cells against MCF-7 cells, we performed cytotoxicity test by co-culturing NK cells with fluorescence-labeled MCF-7 cells. We observed that 2% of dead population in MCF-7 cells was increased up to 7% in the co-culture condition. Interestingly, the proportion of dead cell was significantly raised up to 16–17% when STB-HO was added to co-culture condition (Fig. 7c). Furthermore, the concentration of IFN-γ in co-culture media showed significant elevation following the STB-HO treatment (Fig. 7d).

**Figure 8:**
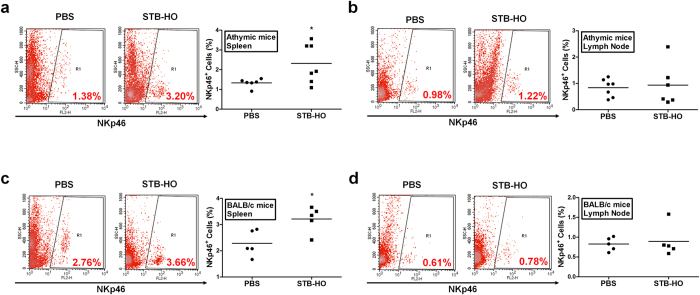
Increase in the number of NK cells residing in mouse spleen by STB-HO administration. (**a–d**) The alteration in the number of NK cells in mice after oral STB-HO administration was determined. The cells from spleen and lymph nodes of (**a,b**) athymic nude mice or (**c,d**) BALB/c mice were isolated and NKp46^+^ cells in isolated cells were detected by flow cytometry. Five to seven mice per group were used. *P < 0.05.

Based on these *in vitro* results showing the NK cell-activating ability of STB-HO, we further investigated whether similar effect can be observed by oral STB-HO administration *in vivo*. To this aim, athymic nude mice and BALB/c mice were orally administered with 70 mg/kg STB-HO daily for 2 weeks, and the population of NKp46^+^ cells was determined. We observed significant increase in NKp46^+^ cells in spleens of both athymic and BALB/c mice while cell population in lymph node was not changed (Fig. 8a–d).

## Discussion

In the present study, we investigated the anti-tumor effect of STB-HO against MCF-7 breast cancer cells. STB-HO administration efficiently suppressed the tumor growth of MCF-7 in xenograft model. These results are consistent with the previous study by Cho *et al*., suggesting the suppressive capability of STB-HO on the growth of HCT colorectal cancer cells^8^. Similarly, a number of studies have provided evidences that minerals have anti-tumor potential in breast cancer cells^32^,^33^. The majority of these studies showed that treatment of minerals including STB-HO on cancer cells lead to the inhibition of cell growth or the induction of apoptosis through the regulation of crucial receptors or signaling. However, in this study, STB-HO treatment didn’t exert direct inhibitory effects on MCF-7 proliferation nor induce the apoptosis while STB-HO suppressed the proliferation of other types of cancer cells. Interestingly, the proliferation of MDA-MB-231, another type of breast cancer cells, was inhibited by the treatment of STB-HO. This discrepancy between two breast cancer cells might result from differences in their nature such as the expression of ER, crucial signaling pathway and the capability to repair DNA. In general, MDA-MB-231 cells are known to be more sensitive to anti-tumor drug treatment, consistent with our findings. However, to precisely explain the underlying mechanisms, the composition of STB-HO is not yet fully discovered. Therefore, a future study to explore the constituents of minerals should be preceded to address this discrepancy in the inhibitory mechanisms. These findings were interesting since STB-HO treatment *in vivo* led to the significant inhibition of tumor growth and down-regulation of growth & metastasis-related signaling in MCF-7 graft model. Instead, STB-HO treatment decreased the expression of HLA class I molecule, a well-known inhibitory ligand for NK cell function, on the surface of MCF-7 cells, indicating that cancer cells exposed to STB-HO might be more susceptible to NK cell cytotoxicity. This suggestion is based on the previously reported studies proposing that various types of tumor cells become susceptible to NK cell-mediated killing when the expression of HLA class I molecules is defective or lower^34^,^35^,^36^. Moreover, STB-HO impaired the secretion of PGE_2_ from MCF-7 cells and inhibited the expression of PGE_2_-producing enzyme, COX-2. Because PGE_2_ is known to contribute to tumor growth, apoptotic resistance, angiogenesis and invasiveness^37^,^38^, one can envision that suppressed production of PGE_2_ by STB-HO treatment can make MCF-7 cells more susceptible to their microenvironment including NK cells.

Mica particles have been continuously developed by several researchers to establish the most effective formulation as feed supplements, and their functions have been explored for application^9^,^10^,^11^,^39^,^40^. The most recent study by Jung *et al*. reported that the newest form of mica, STB-HO, can activate the macrophage which contributes to the immunoenhancement^12^. Consistently, we here demonstrated that STB-HO activates the macrophage by skewing them toward immunostimulatory M1 phenotype, which secretes TNF-α, IL-1β and IL-12. More importantly, since IL-12 signaling is reported to be critical for the effector function of NK cells^41^, the present study found the evidence to associate previous results regarding mica-mediated macrophage activation with anti-tumor effects. Similarly, DCs as well as macrophages produced higher level of IL-12 in response to STB-HO treatment, along with the augmented expression of surface marker for DC activation. Although NK cell is the major component to attack tumor cells, none of previous studies using STB-HO or its prototype explored the influence of mica on NK cell function. We revealed that STB-HO treatment on NK cells generates IFN-γ-secreting effector cells that are capable of killing MCF-7 cells in co-culture system. IFN-γ is a well-known cytokine that polarize macrophages toward M1 type^42^,^43^. Our findings, taken together with previous reports, suggest that NK cells activated by M1 macrophage, in turn, can shape functional behavior of macrophages toward NK stimulatory capability. Since athymic nude mice possess NK cells and macrophages but not T lymphocytes, it seems plausible that our findings of STB-HO-stimulated NK cell cytotoxicity might be a key mechanism for *in vivo* anti-tumor effect. Finally, this study also confirmed that STB-HO administration *in vivo* can actually increase the number of NK cells in spleen of both athymic mice and BALB/c mice. These data support the possibility that STB-HO could be applied for NK cell-mediated immunotherapy against tumor by increasing the absolute number of NK cells, as the insufficient number of NK cells is one of the major impediments for efficient treatment^44^. Although STB-HO has been developed as a feed supplement, securing safety and distribution of the compound is indispensable for clinical use. In this study, no adverse effects were observed in mice during whole period of daily STB-HO administration. Additionally, STB-HO didn’t exert any effects on proliferation and apoptosis of human fibroblasts, representative normal tissue cells. These findings are consistent with the previous study by Cho *et al*., showing that mica particles did not show any cytotoxicity in human umbilical vein endothelial cells as a normal cell line^8^. We further proved that STB-HO didn’t have any harmful effects on human adult stem cells (data not shown). However, we couldn’t uncover the distribution pattern of STB-HO following oral administration into mice, even though we performed several staining techniques using fluorescence or specific staining. Therefore, it is apparent that future work will be required to investigate the distribution and excretion of STB-HO by developing or applying more sensitive techniques of particle detection.

Taken together, the present study revealed novel information implying that mica nanoparticles increase the susceptibility of breast cancer cells to their microenvironment and help microenvironment-constituting NK cells and supporting DCs or macrophages to attack cancer cells. Our findings are expected to provide a basis for development of an alternative therapeutics to treat tumor cells using natural compounds.

## Methods

### Reagents

LPS was purchased from Invivogen (San Diego, CA). IFN-γ was purchased from PeproTech (Rocky Hill, NJ) and IL-12 from R&D Systems (Minneapolis, MN).

### MCF-7 xenograft model

Athymic nude mice (female, 5wk old) were obtained from SLC (Hamamatsu, Japan) and mice were group-housed under specific pathogen-free conditions in the animal facility of Seoul National University. All experiments were performed in accordance with the guidelines and regulation, which were approved by the Institute of Laboratory Animals Resources (SNU-140103-5, Seoul National University, South Korea). 2 × 10^6^ MCF-7 cells mixed with 0.1 mL matrigel (Corning, Corning, NY) were subcutaneously injected into the right flank of athymic nude mice. After one week of stabilization period, the mice were divided into three groups and orally administrated with 35 mg/kg STB-HO (n = 8) or 70 mg/kg STB-HO (n = 7) or normal saline as the positive control (n = 7) daily for 12 weeks. Tumor size was measured twice a week using a caliper. Tumor volume (V) was calculated as the formula: V (mm^3^) = length*width^2^/2. After sacrifice, tumors were isolated for size measurement, histological evaluation and signaling analysis on protein level.

### Histological evaluation

Tumor samples were collected, fixed in 10% formalin, subjected to consecutive steps of alcohol-xylene changes, and embedded in paraffin. Sections of 3 μm thickness were prepared and stained with H&E or aluminum staining.

### Western blot analysis

Tumor samples were minced and lysed in a buffer containing 1% Nonidet-P40 supplemented with a complete protease inhibitor cocktail (Roche, Indianapolis, IN) and 2 mmol/L dithiothreitol. Lysates were resolved by 10% SDS-PAGE, transferred to nitrocellulose membranes, and immunoblotted with the following primary antibodies: rabbit polyclonal anti-ERα (sc-543; Santa Cruz, Dallas, TX), rabbit monoclonal anti-PUMA (12450; Cell Signaling Technology Inc., Danvers, MA), rabbit monoclonal anti-Bak (12105; Cell Signaling Technology Inc.), rabbit polyclonal anti-Cox2 (ab15191; Abcam, Cambridge, UK), rabbit polyclonal anti-EP1 (101740; Cayman, Ann Arbor, MI), rabbit polyclonal anti-EP2 (101750; Cayman), rabbit polyclonal anti-EP3 (101760; Cayman), rabbit polyclonal anti-EP4 (101775; Cayman), mouse monoclonal anti-ß-Actin (3700; Cell Signaling Technology Inc.), mouse monoclonal anti-GAPDH (MAB374; Millipore, Billerica, MA). After immunoblotting with secondary antibodies, proteins were detected with enhanced chemiluminescence (ECL) reagent (RPN2106, GE Healthcare, Buckinghamshire, UK).

### Proliferation assay

The MCF-7 cells (1.5 × 10^6^/well) were seeded in 6-well plates and treated with 10 μg/mL or 50 μg/mL of STB-HO for 72 hours. To calculate the number of viable cells after STB-HO treatment, we performed trypan blue exclusion test, followed by the measurement of protein concentration from lysed cells and the bromodeoxyuridine incorporation assay to confirm the cell proliferation. BrdU proliferation kit was purchased from Roche.

### Apoptosis assay

To evaluate the apoptotic rate, commercially available Apoptosis Detection Kit (BD biosciences, San Jose, CA) was used. After the indicated treatment of STB-HO, the cells were washed twice with PBS and resuspended in 100 μL of binding buffer at a concentration of 1 × 10^5^ and then 5 μL of FITC-conjugated annexin V and 5 μL propidium iodide (PI) were added. The mixtures were gently vortexed and incubated for 15 minutes at room temperature. 400 μL of binding buffer was added to the mixtures, and all samples were analyzed by flow cytometry within an hour, which was performed on a FACS Calibur using the Cell Quest software (BD biosciences).

### Flow cytometric analysis

For the characterization and analysis of markers on cell surface, flow cytometric analyses were performed using a FACS Calibur and the Cell Quest software (BD Biosciences). STB-HO population could be discriminated from MCF-7 cell population on a dot plot image based on the size of particles and cells (Suppl. Fig. S4). MCF-7, DU145, PC3, U87 and MDA-MB-231 cells were incubated with fluorescein isothiocyanate (FITC)-conjugated anti-HLA-ABC antibody (BD Biosciences). Nonspecific isotype-matched antibodies served as controls. For analysis of DC activation, DCs were incubated with Peridinin Chlorophyll (PerCP)-conjugated anti-CD83 antibody (BD Biosciences). NK cells were stained with FITC-anti-CD3 and PE-anti-CD56 antibodies to determine the purity of isolation. Antibodies for NK cell characterization were purchased from BioGems (Westlake Village, CA). All samples were incubated for 30 minutes at room temperature in the dark after Fc receptor blocking.

### Cytokine production

Briefly, cells were treated with STB-HO for 48 hours and culture supernatant was harvested. Production of PGE_2_, IL-10, IL-6 and IL-8 from MCF-7 cells in response to STB-HO was measured in culture media using a commercial enzyme-linked immunosorbent assay kit (R&D Systems). Concentration of TNF-α, IL-1β and IL-12 released from macrophages or DCs as well as NK cell production of IFN-γ were determined by a commercial kit (R&D Systems).

### Differentiation of THP-1 into macrophage-like cells

To generate macrophage-like cells, THP-1 cells (2.5 × 10^5^/mL) were treated with 200 nM phorbol 12-myristate 13-acetate (PMA, Sigma-Aldrich) for 48 hours. Differentiation of PMA treated cells was stabilized by culturing the cells for additional 5 days in fresh media without PMA. Differentiated cells were treated with STB-HO for 2 days and culture media was harvested and determined for cytokine production.

### Isolation and culture of human umbilical cord blood (UCB)-derived mononuclear cell (MNC)

UCB samples were obtained from the umbilical vein immediately after delivery, and the informed consent of the mother was given and approved by the Boramae Hospital Institutional Review Board (IRB) and the Seoul National University IRB (IRB No. 1109/001-006). The UCB samples were mixed with Hetasep solution (StemCell Technologies, Vancouver, Canada) at a ratio of 5:1 and then incubated at room temperature to deplete erythrocyte counts. The supernatant was carefully collected and mononuclear cells were obtained using Ficoll density-gradient centrifugation at 2,500 rpm for 20 minutes. For maintenance of mononuclear cells, the cells were seeded in growth media that consisted of RPMI 1640 (Gibco BRL, Grand Island, NY) containing 10% fetal bovine serum. The cells were further used for the generation of macrophages, DCs and NK cells.

### Generation of macrophages from human MNCs

CD14^+^ monocytes from UCB-derived MNCs were isolated using the immunomagnetic selection via CD14 Microbeads (Milteny Biotech, Auburn, CA) according to the manufacturer’s protocol, yielding an average 98% purity. Briefly, 1 × 10^8^ mononuclear cells were labeled with 200 μL CD14 Microbeads and incubated in isolation buffer for 30 minutes at 4 °C. Cells were washed and resuspended with isolation buffer and the CD14^+^ cells were separated with an LS column (Milteny Biotech) placed on a column adapter in a strong magnetic field. The isolated cells were determined for their purity using flow cytometry (Suppl. Fig. S5) and then gently resuspended in macrophage induction medium, consisting of RPMI1640 medium (Gibco BRL) supplemented with 10% heat-inactivated fetal bovine serum (Gibco BRL), 4 mmol/L GlutaMAX™-I (Gibco BRL). Macrophages were obtained by culturing CD14^+^ monocytes for 7 days in 6-well cell culture plates (Nunc, Roskilde, Denmark) at a density of 1.5 × 10^5^/cm^2^. Cells were refed with fresh culture medium every 3 days. At day 7, the adherent cells were treated with STB-HO for 48 hours and supernatant was harvested and analyzed for cytokine production.

### Generation of DCs from human MNCs

To generate DCs, CD14^+^ monocytes were isolated from UCB-derived MNCs as previously described in macrophage generation. Isolated cells were seeded at a density of 2 × 10^6^ cells/mL in 24-well plates with RPMI1640 (Gibco BRL) media containing 100 ng/mL recombinant human granulocyte macrophage colony-stimulating factor (GM-CSF) (Peprotech, Rocky Hill, New Jersey, USA), 50 ng/mL human recombinant IL-4 (Peprotech) and 10% fetal bovine serum (Gibco BRL). After 3 days, fresh complete media was replaced. On day 5, non-adherent cells were removed and replaced with fresh media containing LPS (1 μL/mL) and IFN-γ (20 ng/mL) or STB-HO to induce DC maturation. To evaluate DC maturation, CD83 expression on the surface of DCs was analyzed by flow cytometry.

### Isolation of NK cells from human MNCs

NK cells were isolated from UCB-derived MNCs using the NK cell isolation kit according to manufacturer’s instruction (#130-092-657, Miltenyi Biotec). Then, isolated NK cells were stained with FITC-anti-CD3 and PE-anti-CD56 antibodies for flow cytometric analysis and further used for NK cell cytotoxicity test.

### NK cell cytotoxicity assay

MCF-7 cells were labeled with CFDA SE Cell Tracer Kit (V12883, Invitrogen, Eugene, OR) and co-culture with isolated NK cells in the presence of STB-HO or IL-12 (0.5 ng/mL) for 4 hours. Then, cells were stained with 2 μg/mL propidium iodide (PI), and PI-incorporated cells among CFDA-labeled cells were detected to analyze the ratio of dead cells in MCF-7 cells. MCF-7 population could be discriminated from NK cell population based on the size, density and fluorescent intensity of cells (Suppl. Fig. S5).

### Detection of NK cells in mouse spleen and lymph nodes

STB-HO (70 mg/kg) was orally administered into athymic nude mice and BALB/c mice for 2 wks. After sacrifice, spleen and lymph nodes were collected. Splenocytes were harvested from spleen by gentle mincing and filtration through the strainer. Cells were isolated from lymph nodes by mincing and subsequent digestion with type IV collagenase (0.5 mg/mL) for 30 minutes at 37 °C. Harvested cells were washed with PBS, followed by the incubation with PE-conjugated anti-NKp46 antibody (BioGems) for 30 minutes at room temperature in the dark. Stained cells were analyzed by flow cytometry.

### Statistical analysis

All experiments were conducted at least three times (n = 3), and the results are expressed as the mean ± SD. All statistical comparisons were made using one-way ANOVA followed by the Bonferroni post hoc test for multigroup comparisons using the GraphPad Prism version 5.01 (GraphPad Software, San Diego, CA). Statistical significance designated as asterisks is indicated in the figure legends.

## Additional Information

**How to cite this article**: Kang, T.-W. *et al*. Mica Nanoparticle, STB-HO Eliminates the Human Breast Carcinoma Cells by Regulating the Interaction of Tumor with its Immune Microenvironment. *Sci. Rep.* **5**, 17515; doi: 10.1038/srep17515 (2015).

## Supplementary Material

Supplementary Information

Supplementary Video 1
