# Supplementary material for: Mica Nanoparticle, STB-HO Eliminates the Human Breast Carcinoma Cells by Regulating the Interaction of Tumor with its Immune Microenvironment
Source: Sci Rep. 2015 Dec 3;5:17515. doi: 10.1038/srep17515 (PMC4668362; doi:10.1038/srep17515)
Supplement: Supplementary Information [file srep17515-s1.pdf]

Supplementary Data for Kang *et al.*

(Title)

Mica Nanoparticle, STB-HO Eliminates the Human Breast Carcinoma Cells by Regulating the Interaction of Tumor with its Immune Microenvironment

Tae-Wook Kang[1,3,†], Hyung-Sik Kim[1,3,†], Byung-Chul Lee[1,2,†], Tae-Hoon Shin[1,2], Soon-Won Choi[1,2], Yoon-Jin Kim[3], Hwa-Yong Lee[3,4], Yeon-Kwon Jung[5], Kwang-Won Seo[2,3,*] and Kyung-Sun Kang[1,*]

[1] Adult Stem Cell Research Center, College of Veterinary Medicine, Seoul National University, Seoul 151-742, South Korea

[2] Research Institute for Veterinary Medicine, College of Veterinary Medicine, Seoul National University, Seoul 151-742, South Korea

[3] Institute for Stem Cell and Regenerative Medicine in Kangstem Biotech, Biomedical Science Building, College of Veterinary Medicine, Seoul National University, Seoul 151-742, South Korea

[4]The Faculty of Liberal Arts, Jungwon University, Chungbuk, 367-805, South Korea.

[5] Seobong BioBestech Co., Ltd., Yeoksam-dong, Kangnam-gu, Seoul, South Korea

[†] These authors contributed equally to this work.

[*]Authors share co-corresponding authorship.

[1]Current affiliation for Hyung-Sik Kim

[4]Current affiliation for Hwa-Yong Lee

Supplementary Methods

Aluminum staining of dissected tumor sections

Paraffin sections were deparaffinized and stained with Einarson's reagent overnight. Stained sections were placed in 0.5% phloxine B for 3min and the excessive dye was removed by washing. Then, the sections were immersed in 5% phosphotungstic acid for 1min and subsequent 1% glacial acetic acid for 2min, followed by washing with 80% ethanol till the sections appear translucent. Sections were further replaced in 1% glacial acetic acid for 1min and counterstained with 0.05% fast green FCF for 3min. After dehydration of the sections with 95% and 100% ethanol (three changes each), coverslips were mounted.

Time-lapse microvideography of prostate cancer cells after STB-HO treatment

Prostate cancer cells (DU145) were treated with STB-HO (10 μg/mL) and sequential photomicrographs were taken every 15 minutes using EVOS® FL Auto (Life Technologies, Carlsbad, CA). Photographs were combined into a movie using the software provided with the instrument (Life Technologies).

Supplementary figure legends

Figure S1. Tracking of STB-HO in tumor tissue.

Aluminum staining was performed to determine the distribution of orally administered STB-HO in tumor tissue. Photographs of stained tumor sections were taken.

Figure S2. Effects of STB-HO treatment on various cancer cells and fibroblasts.

(a) Prostate cancer cells (DU145 and PC3), glioblastoma (U87), breast cancer cells (MDA-MB-231) and human dermal fibroblasts (hDFs) were treated with STB-HO. After 72 hours, a trypan blue exclusion test was performed to verify the proliferation of cells. (b) Proteins from cancer cells and hDFs were analyzed by western blot to identify the expression of apoptosis-related markers.

Figure S3. Effects of STB-HO treatment on the expression of HLA class I in various cancer cells.

The expression of HLA class I on the surface of DU145, PC3, U87 and MDA-MB-231 was detected by flow cytometry after STB-HO treatment.

Figure S4. Gating strategy of flow cytometry to discriminate cells from STB-HO.

The dot-plot population of STB-HO particles, MCF-7 cells and combination of cells with particles was analyzed by flow cytometry. The population in blue and red ellipse indicates STB-HO and MCF-7 cells, respectively.

Figure S5. Characterization of sorted cells and gating strategy for flow cytometry to discriminate MCF-7 cells from NK cells.

(a) To generate macrophages and dendritic cells, $CD14^+$ monocytes were isolated from mononuclear cells and their purity was determined by flow cytometry. (b) Size, density and fluorescence intensity of NK

cells with CFDA-labeled MCF-7 cells were detected by flow cytometry. The population in blue and red ellipse indicates NK cells and MCF-7 cells, respectively.

Supplementary video legends

Video S1. Phagocytosis of STB-HO by prostate cancer cells.

After treatment of STB-HO on prostate cancer cells, a time-lapse photographs were taken by live-imaging system. The photographs were combined into a movie. This movie is representative of independent experiments with other types of cancer cells.

Supplementary Figures

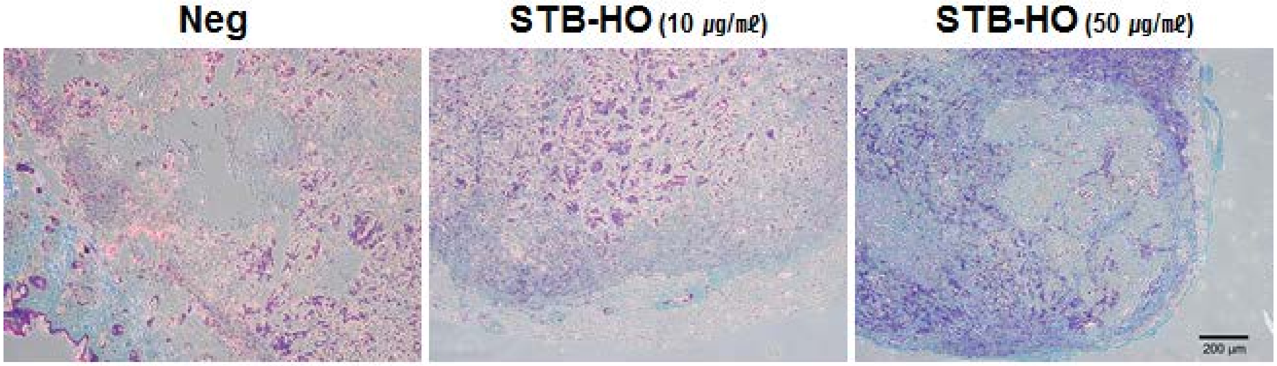

Kang *et al*. Figure S1

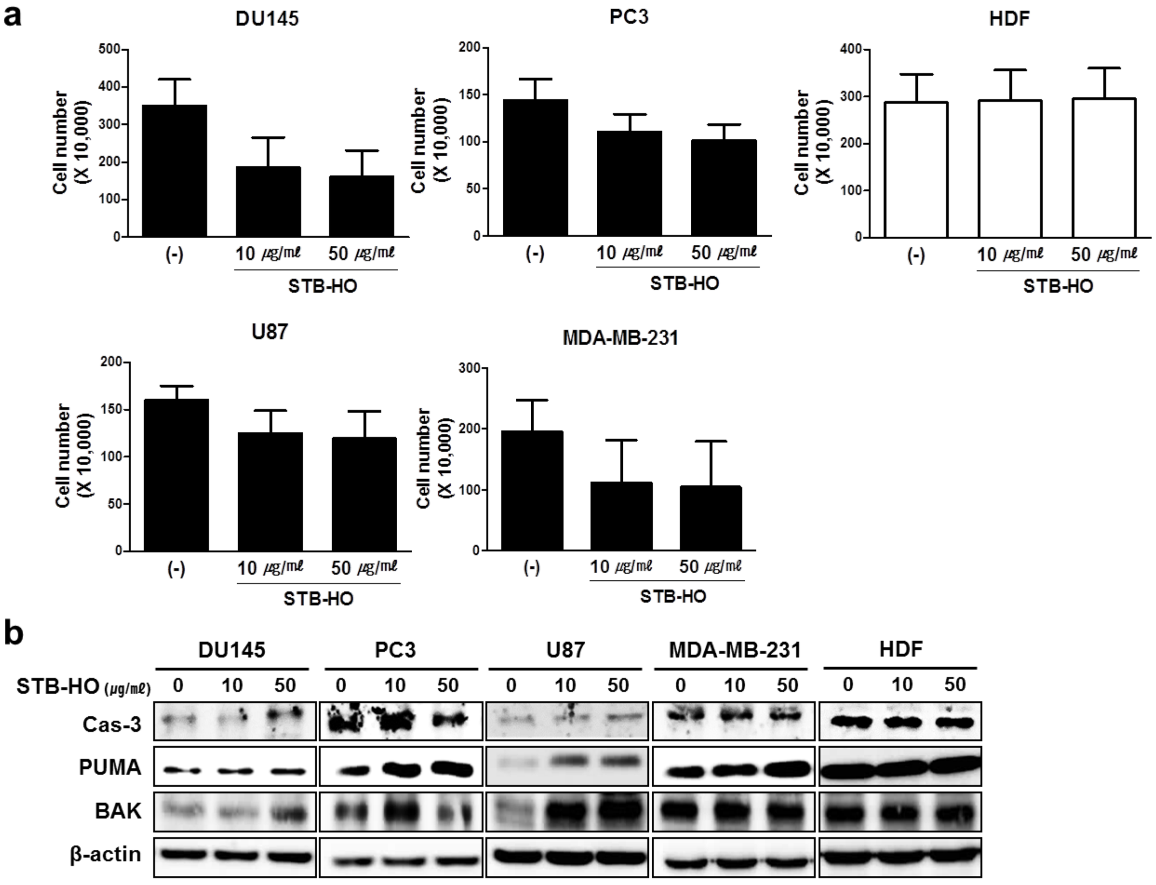

Kang *et al*. Figure S2

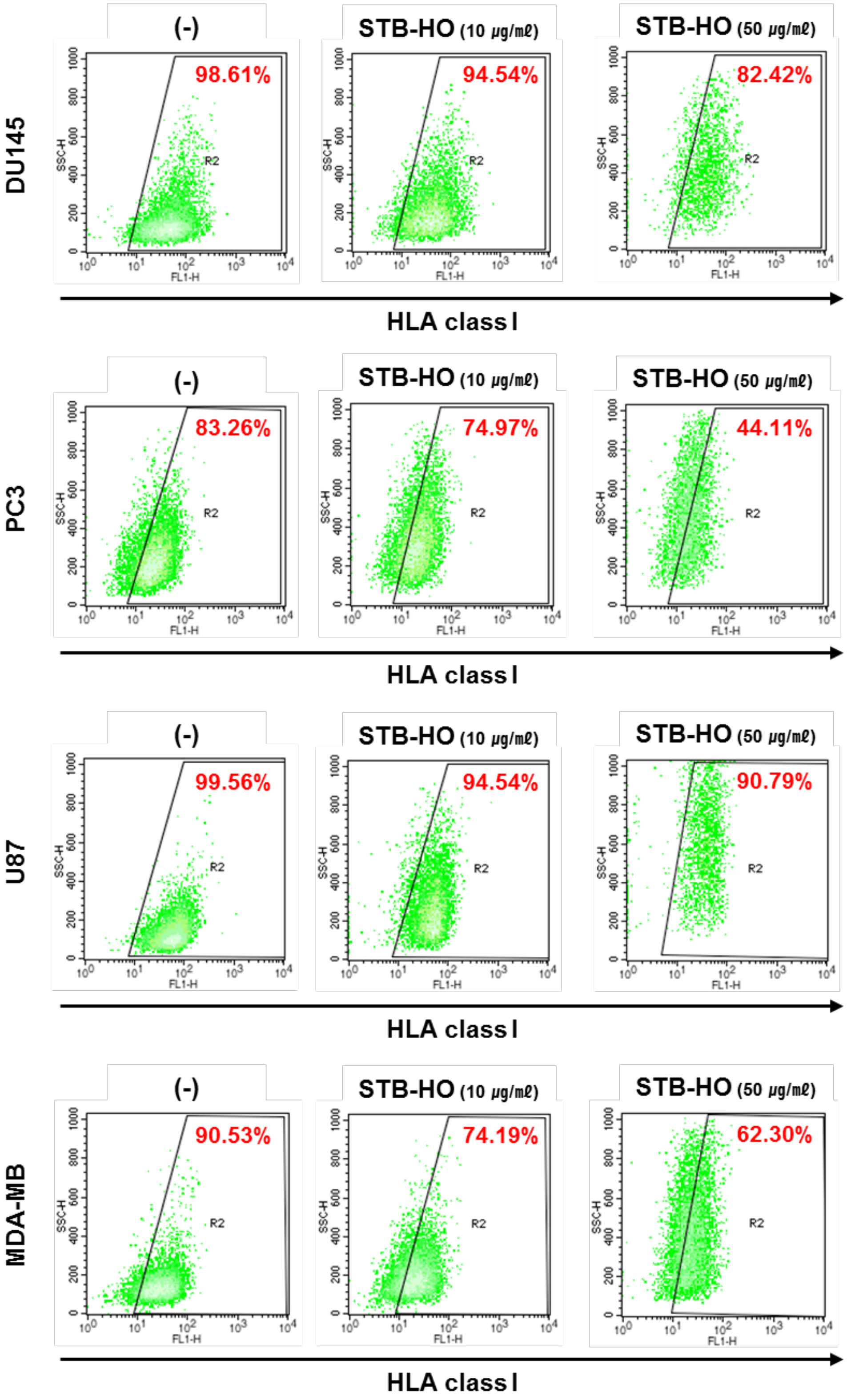

Kang *et al*. Figure S3

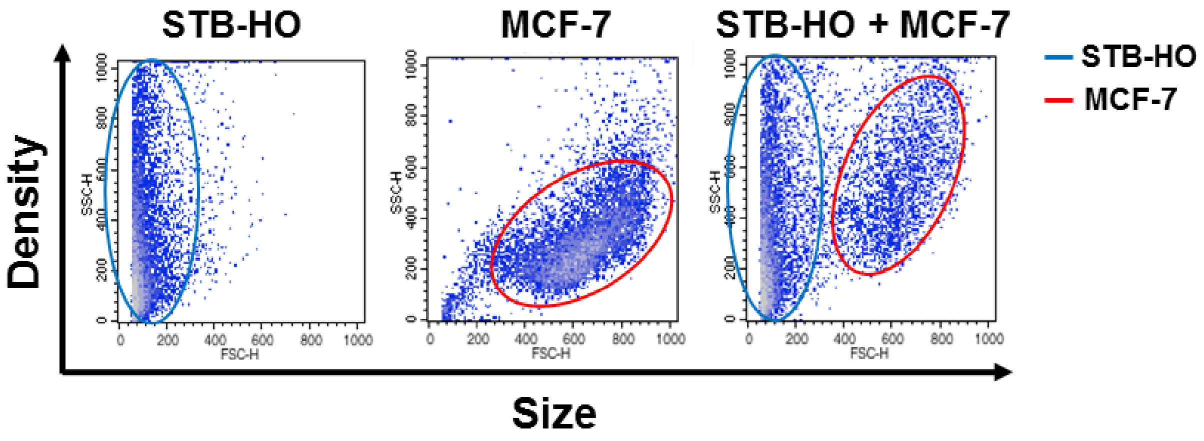

Kang *et al*. Figure S4

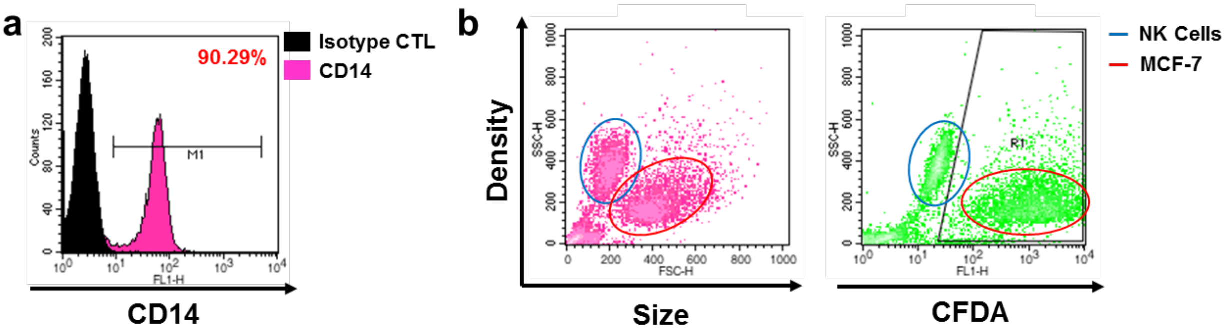

Kang *et al*. Figure S5
